# An Assurance Society

**Published:** 1902-10

**Authors:** 


### AN ASSURANCE SOCIETY.

The need of an assurance organization for the veterinary profession has long been a source of consideration without any definite plans for the same. Whenever a special case among our members finds us without any such society for the protection or support of one of our number the subject again becomes uppermost in our minds. Now, the American Veterinary Medical Association, in convention assembled, has indorsed the project under the strong advocacy of a few members of the Association who have long studied and earnestly advocated the proposition. We would offer as a suggestion to those who will specially consider its feasibility during the ensuing year, that it be started with the American Veterinary Medical Association membership as a basis; that the Association advance the dues to five dollars per year, and that the two dollars be placed as a death benefit fund. That in addition a small charge might be added annually to provide a sick benefit fund; that this fund be measured by the age of the applicants, all unappropriated funds to be set aside as a special emergency fund. The annual dues could be maintained irrespective of one's continuance as a member of the Association, and others could join without membership in any association.

